# Misconceptions About Nested Studies, Matched Analysis, Significancy, Power, Normality and Multivariate Regression Analysis

**DOI:** 10.5812/hepatmon.9052

**Published:** 2013-02-26

**Authors:** Ali Kabir

**Affiliations:** 1 Department of Epidemiology; Faculty of Public Health; Shahid Beheshti University of Medical Sciences, Tehran, IR Iran

**Keywords:** Hepatitis C, Regression Analysis, Case-Control Studies

Dear Editor,

Yan et al. have published a useful paper about thyroid dysfunction (TD) in cases with hepatitis C by treatment with interferon (1). There are some comments which may explicit benefits of this paper in more details. When we report a methodological or statistical issue it is necessary to mention important details which are informative and necessary to know. We should report such details as brief as possible. For example we must mention that type of study is nested case-control or (nested) case-cohort. We should also mention each odds ratio (OR) is crude or adjusted. This study has been nested case-cohort and not nested case-control. In a case-cohort study the comparison group, called a sub-cohort, is selected at random from the initial cohort at baseline regardless of the outcome (2). Nested case-control or case-cohort studies are good approach specifically for evaluating markers which are unknown at the beginning of the cohort or expensive tests. However, when we select cases from the baseline cohort it is case-cohort and when we select simultaneous controls which have similar exposure time with cases in cohort (select controls as the cases are occurring) it is called as nested case-control. Explanation of authors shows that it is a case-cohort study. However, authors declared that it is a nested case-control. It is specifically of importance because when we select our controls among patients of base cohort, there is probability of selecting cases as controls. Since, the prevalence of caseness (having TD) is 11%, there is probability of presence of at least six cases among controls which is considerable and may dilute associations and confound the results. I am interested to know how much is hepatitis C prevalence in southwest of China? They treat approximately 2500 inpatients and 75 000 outpatients per year. Data collection has been done between 2004 and 2011. It seems that there are more than 674 cases under treatment for HCV during this period. How they have selected some of them? In addition, I did not understand that 82 cases are excluded because they either did not meet eligibility criteria or they refused to participate. It seems that they did not have eligibility criteria. However, they have mentioned that participation rate was 87.8% which shows some of cases refused from participation. If the latter is correct, authors should show causes of such non-participation. Were they related to underlying diseases specifically TD or not? Were non-participants significantly different from participants? We should report reasons of non-participation at each stage in such studies (3). Roughly, we need 10 extra cases for including each variable in multiple regression models after first 50 cases which is not attained in this analysis. Moreover, it is not surprising that one thyroid abnormality like positive TPOAb be associated with other thyroid dysfunctions like alteration in TSH, T3 or T4 (total or free component). Finally, if they have calculated OR of positive TPOAb while other non-significant variables were included in the model; this OR is adjusted for these variables and not a crude OR. Authors did not show this is crude or adjusted. In abstract, it has been mentioned that multivariate stepwise regression analysis was used. We should pay attention that when we have more than one dependent variable simultaneously in regression model, it is multivariable. However, in this study multivariate (multiple) and not multivariable analysis has been used. Moreover, stepwise method is pertaining to linear regression and not logistic. Authors have used logistic regression by implementing SPSS which has no option as stepwise for logistic regression. So, they should revise which method has been used instead of stepwise. Authors expressed that controls are sex-and age-matched. Again, there is no more information about type of matching but it seems to be paired matched according to the mentioned results about sex and age description in two groups and other related explanations. When we do pair matching, we need to do conditional logistic instead of ordinary logistic regression. However, if they have selected controls according to frequency match method, using usual logistic regression is correct. T-test is not indicated when you are comparing median. You have shown the median in the Table 1 and did not say in footnote are you comparing mean or not? If you are comparing mean, you may be on right way by using t test. However, for comparing medians, you need another statistical test like sign rank test. Moreover, I am not sure about correct usage of median instead of mean in your study. You mentioned median was used when the distribution was not normal. Interestingly, many authors use only statistical tests like Kolmogorov-Smirnov for checking the normality of distribution. However, statistical tests are misleading without considering graphs most of the times. Figures like Q-Q plot are necessary and helpful for checking normality of distribution and it seems that authors did not consider such issue in checking normality of ALT distribution. Mentioned range is not so consistent with non-normal distribution for ALT. Short telephone interviews have been done in this study. So, this study is at risk of interviewer bias which should be considered and prevented by blinding the interviewer from the situation of being a case or control. Authors did not mention such blinding and does not seem that they have blinded the interviewer because interviewer has asked questions for completing necessary data and most probably has been aware from the situation of cases and controls. Authors have claimed that incidences of developing TD had no significant difference for each of the HCV genotypes. When your sample size in subgroups is low, non-significances have limited importance. Power is more useful index showing the importance of your finding in such situations. Correctly, when you merge your similar groups (for example genotypes 2, 3 and 6) with each other and compare them with genotype 1, you increase your power. They have mentioned that mean follow-up period was 36 months. It is better to mention median (instead of mean) for time which is a variable without normal distribution.
